# Quantitative proteomic analysis and functional characterization of *Acanthamoeba castellanii* exosome-like vesicles

**DOI:** 10.1186/s13071-019-3725-z

**Published:** 2019-10-09

**Authors:** Wei-Chen Lin, Chia-Yun Tsai, Jian-Ming Huang, Shang-Rung Wu, Lichieh Julie Chu, Kuo-Yang Huang

**Affiliations:** 1 Department of Microbiology and Immunology, College of Medicine, National Cheng Kung University, Tainan City, 701 Taiwan; 2 Department of Parasitology, College of Medicine, National Cheng Kung University, Tainan City, 701 Taiwan; 3 Institute of Basic Medical Sciences, College of Medicine, National Cheng Kung University, Tainan City, 701 Taiwan; 4 Institute of Oral Medicine, College of Medicine and Hospital, National Cheng Kung University, Tainan City, 701 Taiwan; 5 Molecular Medicine Research Center, College of Medicine, Chang Gung University, Taoyuan City, 333 Taiwan; 6 Liver Research Center, Chang Gung Memorial Hospital at Linkou, Gueishan, Taoyuan City, 333 Taiwan; 7 Graduate Institute of Pathology and Parasitology, National Defense Medical Center, Taipei City, 114 Taiwan

**Keywords:** *Acanthamoeba*, Extracellular vesicles, Exosome

## Abstract

**Background:**

Pathogenic protozoans use extracellular vesicles (EVs) for intercellular communication and host manipulation. *Acanthamoeba castellanii* is a free-living protozoan that may cause severe keratitis and fatal granulomatous encephalitis. Although several secreted molecules have been shown to play crucial roles in the pathogenesis of *Acanthamoeba*, the functions and components of parasite-derived EVs are far from understood.

**Methods:**

Purified EVs from *A. castellanii* were confirmed by electron microscopy and nanoparticle tracking analysis. The functional roles of parasite-derived EVs in the cytotoxicity to and immune response of host cells were examined. The protein composition in EVs from *A. castellanii* was identified and quantified by LC-MS/MS analysis.

**Results:**

EVs from *A. castellanii* fused with rat glioma C6 cells. The parasite-derived EVs induced an immune response from human THP-1 cells and a cytotoxic effect in C6 cells. Quantitative proteomic analysis identified a total of 130 proteins in EVs. Among the identified proteins, hydrolases (50.2%) and oxidoreductases (31.7%) were the largest protein families in EVs. Furthermore, aminopeptidase activities were confirmed in EVs from *A. castellanii*.

**Conclusions:**

The proteomic profiling and functional characterization of EVs from *A. castellanii* provide an in-depth understanding of the molecules packaged into EVs and their potential mechanisms mediating the pathogenesis of this parasite.

## Background

*Acanthamoeba* spp. are free-living amoeba and distributed in diverse natural environments, including water and soil. *Acanthamoeba* is capable of causing a rare but fatal encephalitis known as granulomatous amoebic meningoencephalitis (GAE) [1] or a painful and sight-threatening corneal infection termed *Acanthamoeba* keratitis (AK) [2]. GAE is associated with immunocompromised individuals [3], whereas the patients affected by AK are generally immunocompetent [4]. AK is also a rare disease, with less than 150 reported cases each year in the USA [5], and this may be due to the significant role of the mucosal immune system [6]. GAE has led to a mortality rate of more than 90% due to the lack of specific anti-acanthamoebic drugs and the selectivity of the blood-brain barrier [7]. Additionally, *Acanthamoeba* has been considered a Trojan horse for other microbial pathogens, including a variety of viruses, bacteria, and fungi [8]. Hence, it is essential to further elucidate the mechanism of *Acanthamoeba* spp. pathogenesis and develop chemotherapeutic agents to combat *Acanthamoeba* infections.

The host is probably affected by the release of soluble mediators by parasites that degrade or interact with their target cells. *Acanthamoeba* spp. possess hydrolytic enzymes, including elastases [9], phospholipases [10], glycosidases and a variety of serine, cysteine and metalloproteases [11, 12], and some of these extracellular protease activities are increased in pathogenic *Acanthamoeba* strains [13, 14]. For example, mannose-induced 133 kDa protease (MIP133) and aminopeptidase M20/M25/M40 family protein have been identified as the principal virulence factors of pathogenic *A. castellanii* [15, 16]. Extracellular vesicles (EVs) that are secreted into the extracellular milieu have emerged as key players in intercellular communication without direct cellular contact. EVs can be mainly classified into exosomes and ectosomes [17]. Exosomes are 30–100 nm membrane-bounded vesicles generated within multivesicular bodies (MVBs) that are specialized late endosomes and can traffic to the plasma membrane, with which they fuse to release exosomes. Ectosomes range from 100 to 500 nm in diameter and are produced *via* budding of the plasma membrane. Exosomes contain various molecular constituents of the original cell, including proteins, mRNAs, and microRNAs [18].

The production of exosomes has been described in parasites, and the importance of these EVs during host-parasite interactions has been highlighted [19–33]. In a previous study, the pro-inflammatory (interleukin-6 [IL-6], IL-12) and anti-inflammatory (IL-10) cytokines in monocytes/macrophages were shown to be elevated by stimulation with *Acanthamoeba*-derived cell-free conditioned medium [34]. In addition to the secreted proteins mentioned above, the EVs of *A. castellanii* have been reported to be cytotoxic to epithelial and glioblastoma cells [33, 35]. Hence, it is likely that the EVs of *Acanthamoeba* spp. not only play a crucial role in the progression of infection but also elicit the host immune response.

In the present study, we demonstrate that *A. castellanii* secretes EVs with physical characteristics similar to mammalian and other protozoan exosomes. The roles of EVs from *A. castellanii* in the host cell immune response and pathogenicity are validated. The protein composition of EVs from *A. castellanii* is quantified by a LTQ-orbitrap-based proteomic approach, which sheds light on the potential contact-independent mechanism mediating the pathogenesis of this parasite.

## Methods

### Cell culture

*Acanthamoeba castellanii* (ATCC 30010) was axenically cultured in a proteose peptone yeast extract glucose (PYG) medium, pH 6.5, at 28 °C in cell culture flasks. Trophozoites were harvested at the logarithmic growth phase after cultivation for 3–5 days. Rat glial C6 cells (ATCC CCL-107) were cultured in 10 ml Dulbecco’s minimal essential medium (DMEM) supplemented with 5% heat-inactivated fetal calf serum at 37 °C and 5% CO_2_. THP-1 cells (ATCC TIB-202) were maintained in continuous culture in RPMI 1640 medium supplemented with 10% heat inactivated fetal calf serum, 100× l-glutamine (Caisson Labs, Utah, USA), 100× antibiotic-antimycotic (Caisson Labs, Utah, USA) at 37 °C and 5% CO_2_.

### Isolation of EVs from *Acanthamoeba castellanii*

*Acanthamoeba castellanii* was cultured in PYG medium in T-75 flasks until they reached 80% confluency, followed by removal of the culture medium. The cells were washed three times and resuspended in Pageʼs modified Neffʼs amoeba saline (PAS) (120 mg NaCl, 4 mg MgSO_4_ × 7H_2_O, 3 mg CaCl_2_, 142 mg Na_2_HPO_4_, 136 mg KH_2_PO_4_ in 1 l distilled water) for 48 h. The cell debris was removed by centrifugation at 2000×*g* for 30 min and further centrifuged at 10,000×*g* for 30 min. The cell-free medium was ultra-filtered through Amicon ultracentrifugation filters (Merck Millipore, Billerica, MA, USA), then filtered through a 0.22 μm filter. Next, 0.5 volumes of the total exosome isolation reagent (Life Technologies, Carlsbad, CA, USA) were added to the supernatants and incubated at 4 °C overnight. After incubation, the supernatants were centrifuged at 10,000×*g* for 1 h. Pellets were resuspended in 50 μl PBS and stored at − 80 °C. The concentration of exosome protein was determined by the Bradford assay (Bio-Rad Laboratories, Inc., USA). The protein samples were separated with 10% SDS-PAGE (T-Pro, Taipei, Taiwan) followed by silver staining using standard procedures.

### Internalization of EVs from *A. castellanii* with rat glial C6 cells

Purified EVs were labeled with PKH67 Green Fluorescent Cell Linker kit (Sigma-Aldrich, St. Louis, USA) according to the manufacturer’s instructions with modification. Briefly, EVs were mixed in 150 μl of PBS with an equal volume of diluent C and PKH67 dye at room temperature for 3 min in the dark. The reaction was terminated by adding 0.1% bovine serum albumin (BSA). The PKH67-labeled EVs were then centrifuged at 14,000× *rpm* for 1 h at 4 °C. Pellets were collected and washed with PBS at 14,000× *rpm* for 30 min at 4 °C. The PKH67-labeled vesicles were obtained and resuspended in PBS. For fluorescence microscopy analysis, 1 × 10^5^ C6 cells/ml were seeded on an 18 × 18 mm coverslip at 37 °C for 24 h in a 5% CO_2_ incubator. After incubation, cells were washed with PBS and co-incubated with PKH67-labeled EVs at different time points to observe the internalization event after short and prolonged incubation (15 min, 30 min, 1 h, 2 h, 4 h and 8 h). The cells were washed three times with PBS, fixed with 4% paraformaldehyde for 30 min and then stained with 4,6-diamidino-2-phenylindole (DAPI) as a counterstain before being mounted with Fluoromount™ Aqueous Mounting Medium (Sigma-Aldrich). The internalization of EVs by cells was visualized with fluorescence microscopy (Olympus Cell^R^, Olympus Corp., Tokyo, Japan).

### Assay of cytopathic effects (CPE)

The rat glial C6 cells (1 × 10^5^ C6 cells/ml) were cultured in a 6-well plate for 24 h. After removing the culture medium, the cells were washed with PBS. C6 cells were cultured in DMEM without FBS (500 μl) and incubated with *Acanthamoeba* EVs (100 μg) in PBS. The total volume of culture medium was adjusted to 1 ml. DMEM without FBS (500 μl) combined with PBS (500 μl) served as a negative control. After 24 h of co-incubation, the cells were washed, paraformaldehyde-fixed, and then stained with Giemsa stain (Merck, Darmstadt, Germany) [36].

### Live-cell imaging of C6 cells coincubation with EVs from *A. castellanii*

The live-cell image analysis was conducted by microscopy (Olympus Cell^R^) as previously described [15]. The morphology of rat glial C6 cells was observed after coincubation with EVs for 24 h.

### Total RNA isolation and cDNA synthesis

RNA was isolated using the Total RNA Extraction Miniprep System (Viogene, CA, USA). RNA was stored at − 70 °C. The concentration and A260/A280 ratio of RNA were measured with the ND-1000 (NanoDrop, Thermo, Waltham, MA, USA).

cDNA synthesis was performed using the High Capacity cDNA Reverse Transcription Kit (Applied Biosystems, CA, USA). The kit components were allowed to thaw on ice and 20 µl reaction volumes were prepared according to the manufacturer’s instructions. Reverse transcription conditions were set as follows: 25 °C for 10 min, 37 °C for 120 min, 85 °C for 5 min, and a hold at 4 °C.

### Reverse transcription-PCR

One-step reverse transcription PCR (RT-PCR) was performed with SuperScript™ One-Step RT-PCR with the Platinum Taq kit (Thermo Fisher Scientific, CA, USA) to investigate gene expression. All of the cDNA was synthesized from 1 μg of total RNA from THP-1 cells. RT-PCR products were separated by electrophoresis on a 1% agarose ethidium bromide (EtBr) stained gel The primers used in this study are listed in Additional file 1: Table S1.

### In-sol digestion of EV proteins

A total of 10 μg exosome protein was reconstituted with 50 mM ammonium bicarbonate, reduced with 5 mM dithiothreitol (Merck, Darmstadt, Germany) at 56 °C for 45 min, and blocked cysteine with 10 mM iodoacetamide (Sigma-Aldrich) at 25 °C for 30 min. All samples were digested with trypsin (Promega, Madison, WI, USA) at a 1:25 enzyme:substrate ratio at 37 °C for 16 h. Then, the digested peptides were desalted and dried for storage at − 80 °C until use.

### LC-MS/MS analysis for EV protein identification

The dried peptide mixtures were reconstituted with 0.1% formic acid for analysis using a nanoLC-LTQ-Orbitrap hybrid mass spectrometer (Thermo Fisher Scientific, San Jose, CA, USA) as described previously [37]. The MS raw data files were analyzed by Proteome Discoverer Software (version 1.4, Thermo Fisher Scientific) and searched against the UniProt database (downloaded on 15 Nov 2017, extracted for *A. castellanii*; 17,223 sequences) using the Mascot search engine (Matrix Science, London, UK; version 2.5). One missed cleavage was allowed for trypsin digestion, 10 ppm mass tolerance was permitted for peptide masses, and 0.5 Da was chosen for CID fragment ions for peptide identification. The settings of fixed and variable modifications were carbamidomethyl and oxidized methionine/acetyl, respectively. Peptide-spectrum matches (PSMs) were then filtered based on high confidence and rank 1 of peptide identification in the Mascot search to ensure an overall false discovery rate below 0.01. Proteins with a single peptide hit were removed.

### Aminopeptidase activity assay

The aminopeptidase activity in EVs was measured by the hydrolysis of l-leucine-7-amido-4-methylcoumarin hydrochloride (Leu-AMC) and l-arginine-AMC (Arg-AMC) (Sigma-Aldrich) as previously described [15]. Briefly, the EV solution was added to assay buffer (50 mM Tris-HCl, pH 8.0) containing 10 μM Leu-AMC or Arg-AMC, followed by incubation at 37 °C for 30 min. The PBS buffer served as the control for this assay. The release of fluorescence was detected at an excitation wavelength of 370 nm and an emission wavelength of 440 nm using a FlexStation 3 Multi-Mode microplate reader (Molecular Devices, Sunnyvale, CA, USA) [38].

### Transmission electron microscopy

Isolated EVs were added to charged carbon-coated grids after being stained with 1% uranyl acetate. EVs treated with uranyl acetate were examined using a transmission electron microscope (JEM-1400, Jeol Ltd., Tokyo, Japan).

### Statistical analysis

Quantitative data were expressed as mean ± SD. of three independent experiments. Studentʼs t-test (two-tailed) was used to evaluate the significant differences between groups. *P* < 0.05 was considered statistically significant.

## Results

### Physical characterization of EVs produced by *A. castellanii*

**Fig. 1:**
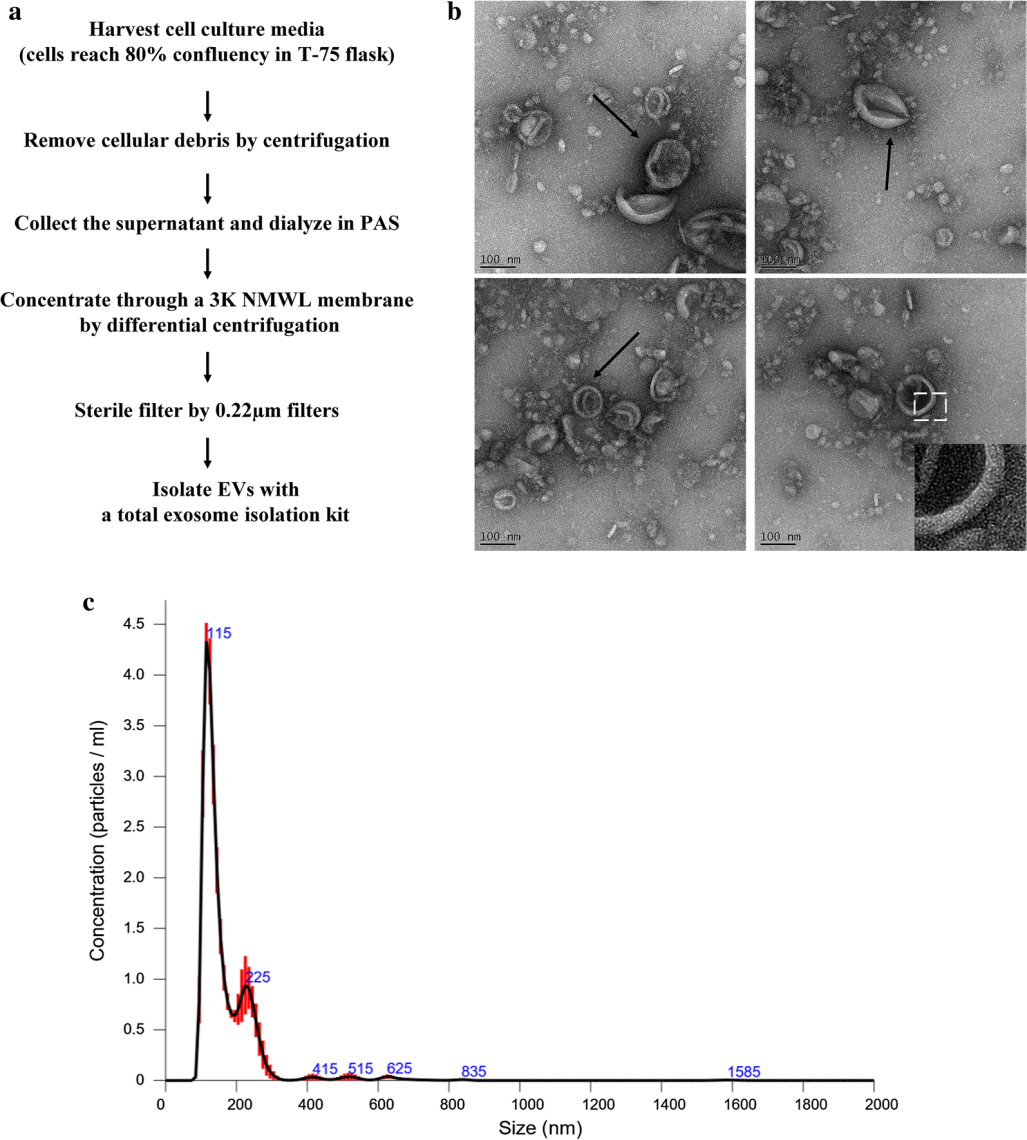
Isolation and physical characterization of EVs from *Acanthamoeba castellanii*. **a** The flow chart of EV isolation from the excretory-secretory products of *A. castellanii*. **b** Morphological characterization of EVs by TEM. Arrows indicate isolated EVs stained with uranyl acetate. The enlarged region shows the EVs with a double membrane structure. **c** Representative nanoparticle tracking analysis (NTA) plot showing the distribution of size and concentration of EVs

To test whether *A. castellanii* produces EVs, we isolated the fraction from cell-free medium that contained secreted proteins, followed by a series of centrifugation and concentration steps. The commercial exosome isolation reagent was used to extract the less soluble components, such as vesicles (Fig. 1a). The purified fraction of EVs was confirmed by electron microscopy (EM) (Fig. 1b) and nanoparticle tracking analysis (NTA) (Fig. 1c). EM analysis revealed round or cup-shaped vesicles with double membranes, similar to mammalian and other protozoan exosomes. NTA identified a major population of vesicles, with a concentration of 2.83 × 10^10^ (particles/ml) and a mean diameter of 166.7 nm. Among them, a large number of vesicles were ~ 118 nm in size. This solubility-based approach yielded purified EVs from *A. castellanii* with characteristics similar to previously identified exosomes [39].

### EVs from *A. castellanii* are internalized into rat glial C6 cells

**Fig. 2:**
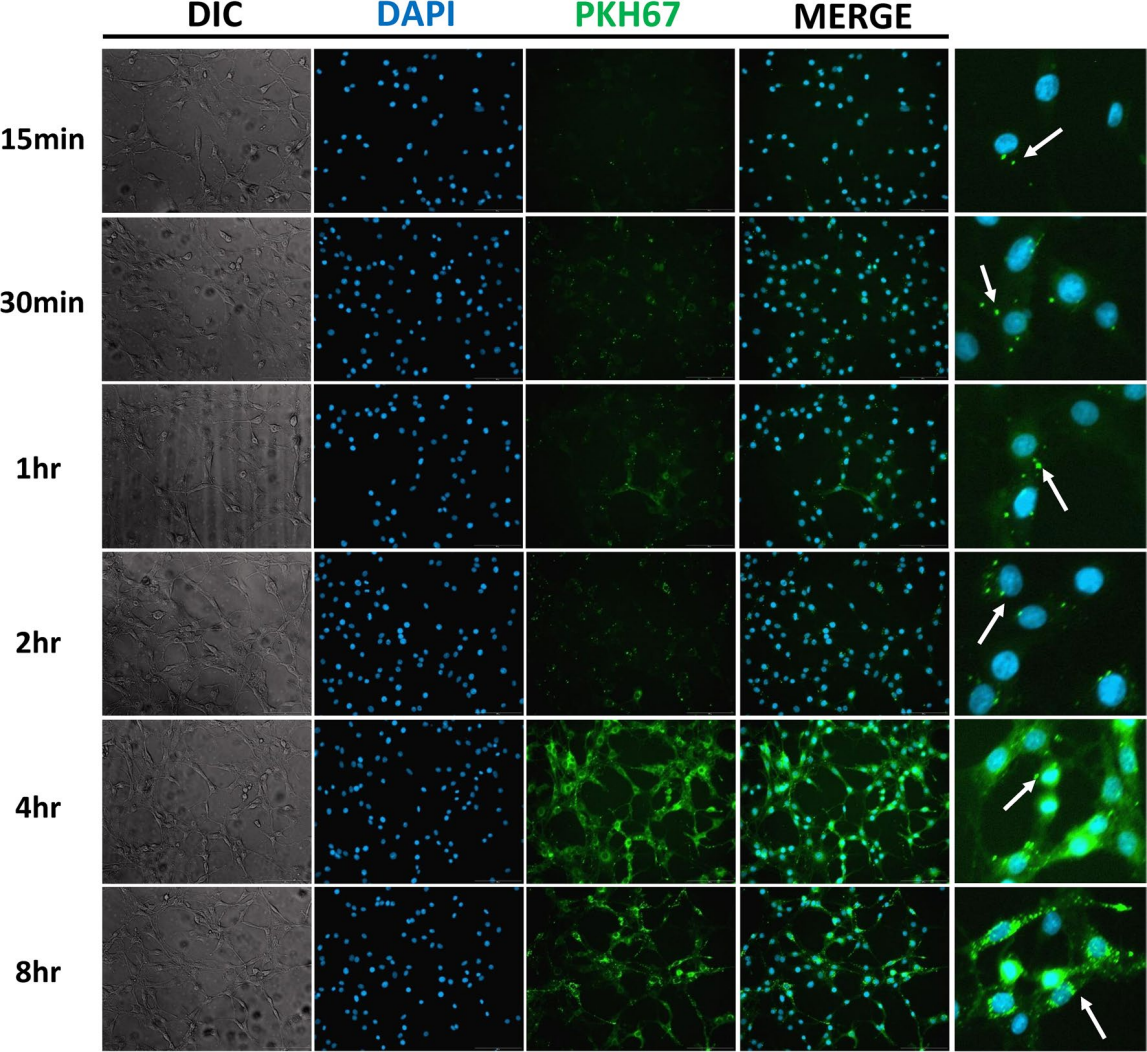
Uptake of PKH67-labeled EVs by C6 cells. Rat glial C6 cells were cocultured with PKH67-stained EVs (green) from *Acanthamoeba castellanii* and fixed for fluorescence microscopy. C6 cells were incubated with PKH67-labeled EVs for 15 min, 30 min, 1 h, 2 h, 4 h or 8 h. Nuclei were stained with DAPI (blue). Arrows indicate EVs that were taken up by C6 cells

Increasing evidence suggests that protozoan exosomes are able to mediate cell-cell communication and host cell response by delivering their contents into host cells [17]. We thus examined whether EVs from *A. castellanii* can fuse with their target cells by labeling the membrane of EVs with PKH67 fluorescent dye, followed by incubation with rat glial C6 cells for different time intervals (15 min, 30 min, 1 h, 2 h, 4 h and 8 h). The punctate signals of PKH67-labeled EVs were strongly observed in C6 cells after short coincubation (15 min) and increased in a time-dependent manner (Fig. 2). After prolonged incubation (4 h or 8 h) with EVs, the dispersed fluorescence in the cytoplasm of C6 cells suggested that EVs had delivered their contents into their target cells. This result clearly indicates that EVs of *A. castellanii* are internalized into rat glial C6 cells.

### EVs from *A. castellanii* lead to cytotoxic effects on rat glial C6 cells

**Fig. 3:**
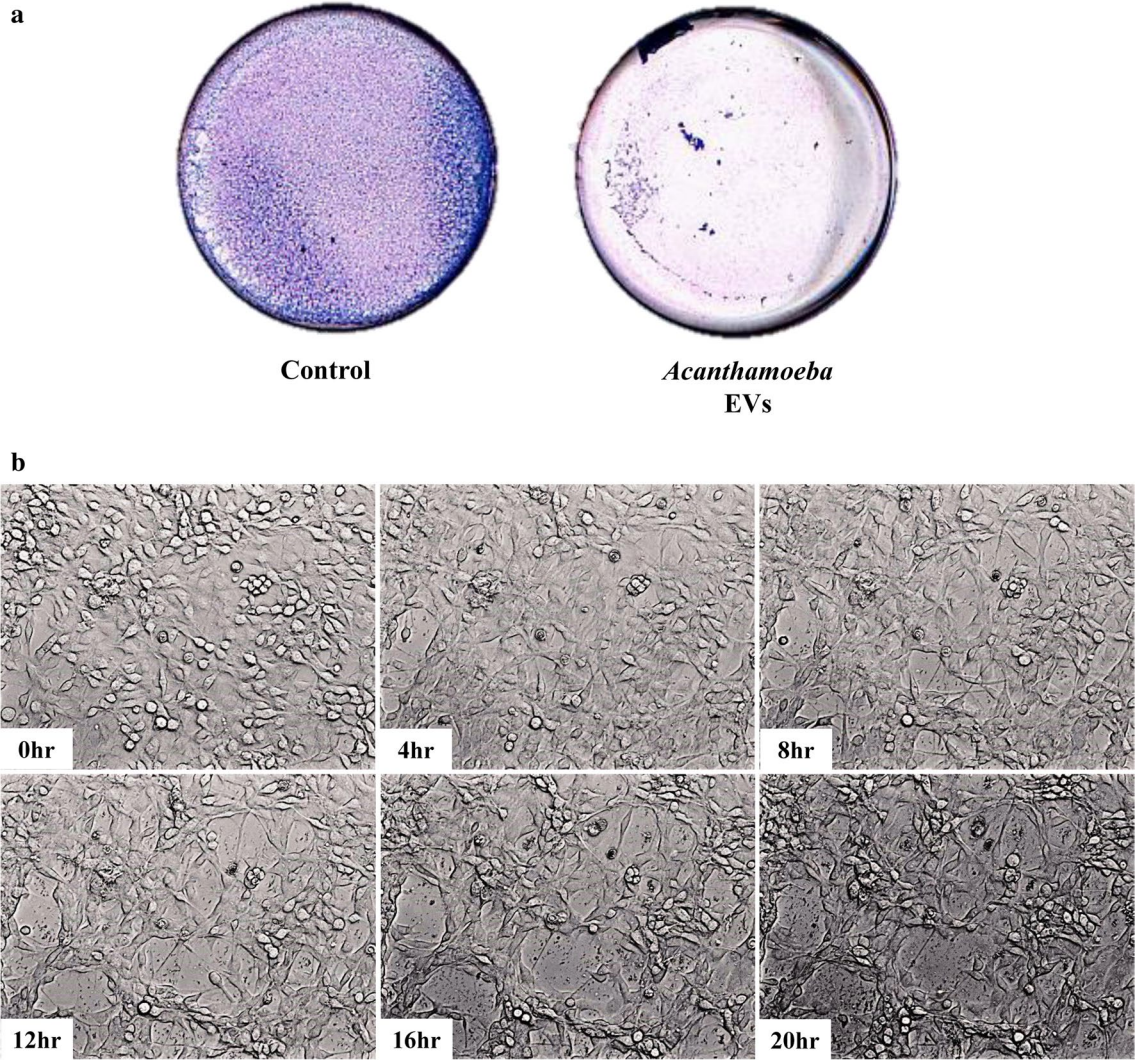
Cytopathogenicity of EVs from *Acanthamoeba castellanii* on C6 cells. **a** Rat glial C6 cells were cocultured with EVs from *A. castellanii*, and the CPE was evaluated by Giemsa staining after cocultivation for 24 h. The data are representative of three independent experiments. **b** Light micrographs show the CPE of C6 cells produced by EVs from *A. castellanii* after cocultivation for 4 h, 8 h, 12 h, 16 h and 20 h

The cytopathic effect (CPE) assay was done to evaluate the effect of C6 cell coincubation with different *Acanthamoeba* strains [36]. We previously demonstrated that the pathogenic strain NCKH_D causes C6 cell damage, which is mediated by both contact-dependent and contact-independent destruction [15]. *Acanthamoeba* secreted proteins (Asp) play an important role in the contact-independent cytolysis of C6 cells. To examine whether EVs of *A. castellanii* also lead to cytotoxic effects on C6 cells, the CPE of C6 cells was evaluated after co-culture with EVs from *A. castellanii* for 24 h. The data showed that C6 cells markedly detached from the culture plates after coincubation with EVs from *A. castellanii* compared with the PBS-treated control, suggesting that parasite-derived EVs destroyed the adhesive ability of target cells and caused cell death (Fig. 3a). Time-lapse microscopy analysis also revealed that the C6 cells were more spherical and cytolytic following exposure to EVs from *A. castellanii* (Fig. 3b). These results provide *in vitro* evidence that EVs from *A. castellanii* disrupt mammalian host cells during the establishment of an infection.

### EVs from *A. castellanii* induce the immune response of human THP-1 cells

**Fig. 4:**
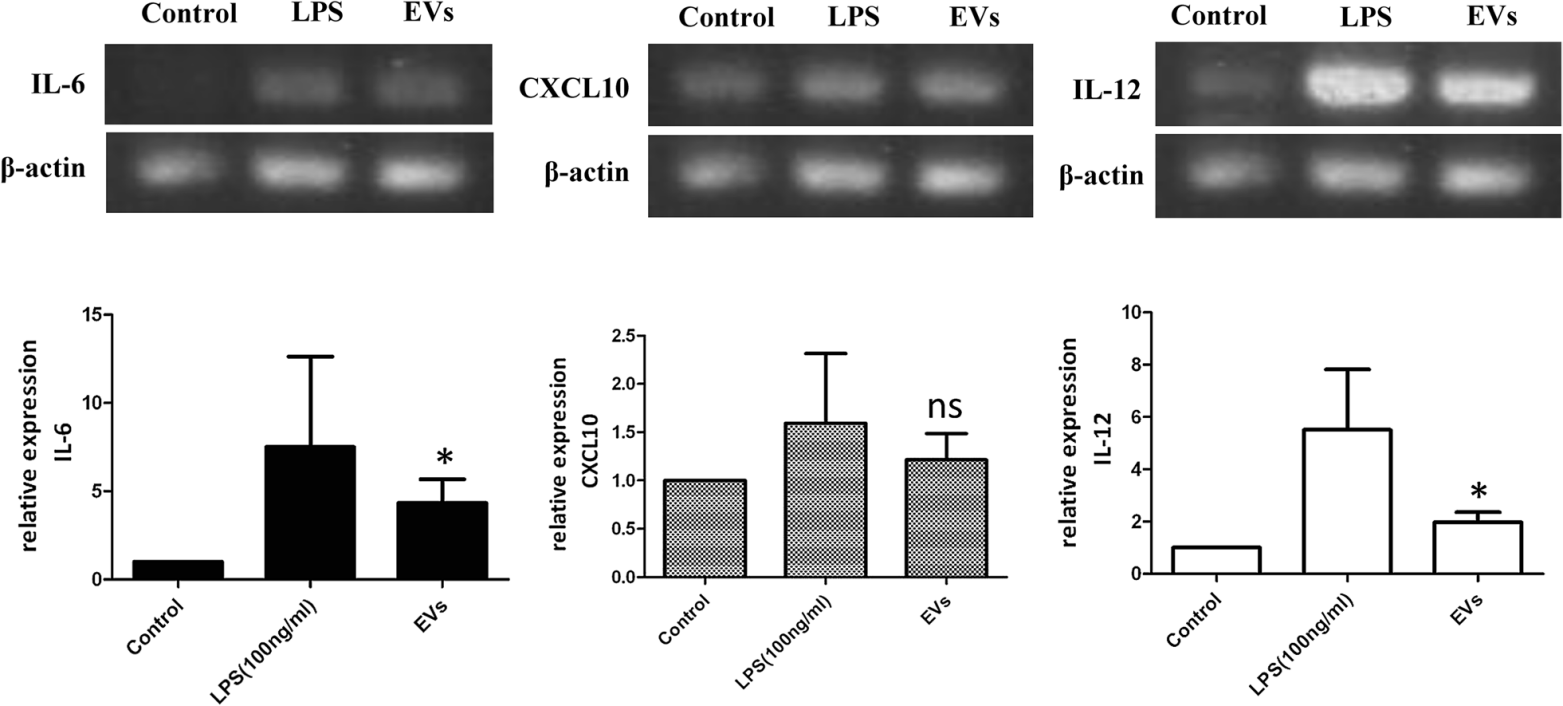
EVs from *Acanthamoeba castellanii* trigger the expression of IL-6 and IL-12 by THP-1 cells. THP-1 human monocytic cells were stimulated with EVs from *A. castellanii*, lipopolysaccharides (LPS) or PBS for 24 h. LPS and PBS served as positive and negative controls for cytokine stimulation, respectively. The mRNA expression levels of IL-6, IL12, CXCL10 were examined by RT-PCR. The results were obtained from three independent experiments and are represented as the mean ± SD. β-Actin was used as an internal control for RT-PCR. *P < 0.05; ns, not significant

In addition to *Acanthamoeba* trophozoites, amoeba-derived cell-free conditioned medium has been found to stimulate the production of proinflammatory cytokines, such as tumor necrosis factor alpha (TNF-α), IL-6, and IL-12 [34]. To clarify the effect of EVs from *A. castellanii* on the host immune response, we detected the mRNA expression levels of cytokines IL-6, IL-12, and CXCL10 secreted by human monocytic THP-1 cells in response to EVs. The data showed that EVs from *A. castellanii* triggered an immune response in THP-1 cells, resulting in increased transcription of the proinflammatory cytokines IL-6 and IL-12 (*P* < 0.05) (Fig. 4). There was no significant change in CXCL10 transcription after exposure to EVs from *A. castellanii*. Similar to the observations reported in other pathogenic protozoans [24, 40], we demonstrate for the first time that EVs from *A. castellanii* send a message to immune cells and elicit an immune response.

### Quantitative proteomic analysis of EVs from *A. castellanii*

**Fig. 5:**
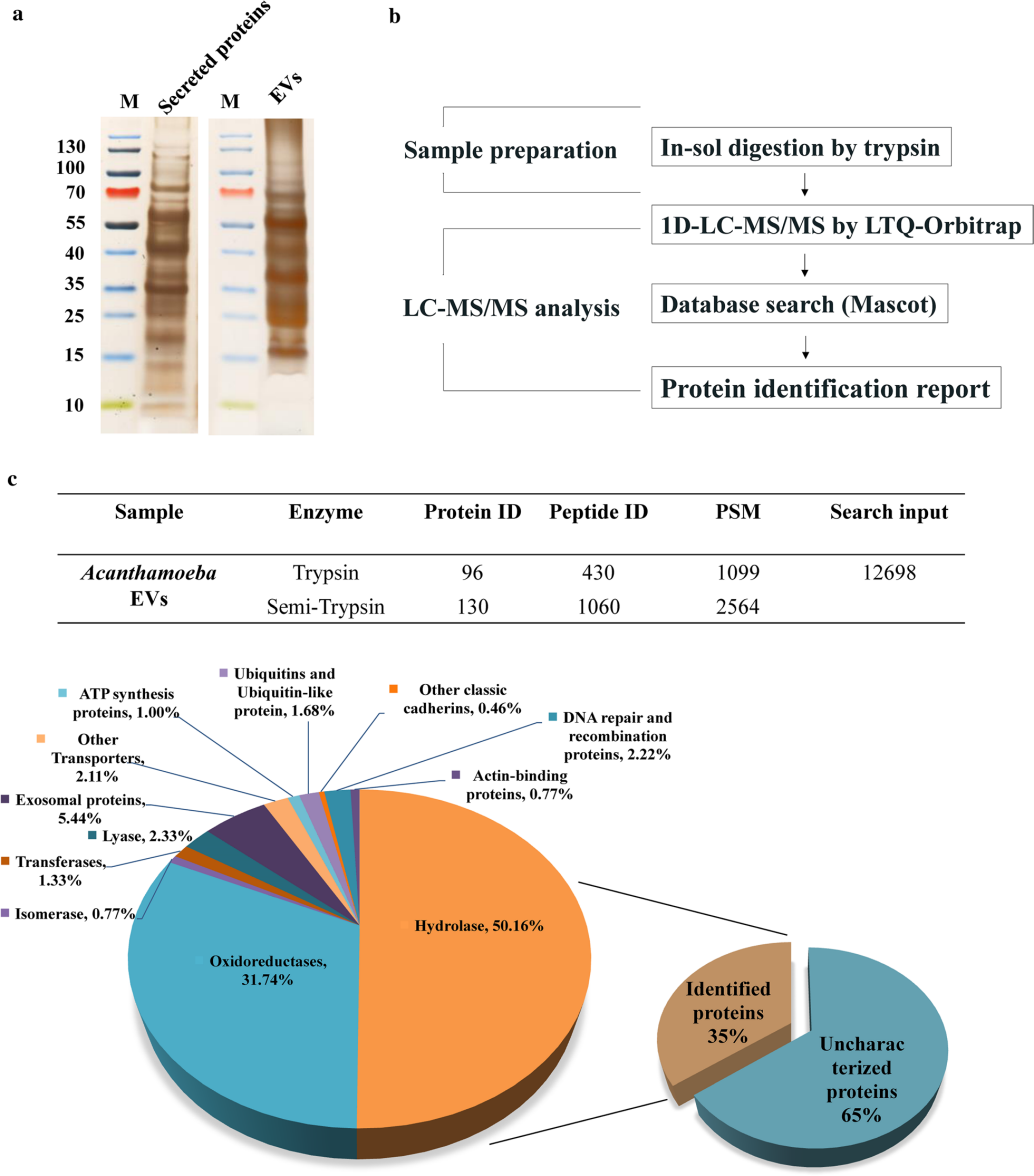
Proteomic analyses of EVs from *Acanthamoeba castellanii*. **a** The secreted proteins and the EV fraction harvested from *A. castellanii* culture medium were analyzed by SDS-PAGE and silver staining. Lane M: Protein marker. **b** The workflow for proteomic profiling of EVs. **c** Identification and functional classification of proteins in EVs from *A. castellanii*

To better understand the components of EVs in *Acanthamoeba*, proteins collected from the fraction of EVs from *A. castellanii* were analyzed by SDS-PAGE followed by silver staining. The pattern of protein bands separated from the EV fraction was not similar to that of the fraction of secreted proteins (Fig. 5a), suggesting that the distinct proteins were packaged into EVs from *A. castellanii*. To determine the protein composition in EVs, the proteins were analyzed by 1D-LC-MS/MS using an LTQ-Orbitrap mass analyzer (Fig. 5b). A total of 130 proteins were identified from EVs of *A. castellanii* (Additional file 2: Table S2). The identified proteins were classified into several functional groups based on KEGG annotations (Fig. 5c). Approximately 65% of the proteins in EVs were previously uncharacterized. Among the identified proteins with functional annotations, the largest protein families in EVs were categorized into hydrolases (50.2%) and oxidoreductases (31.7%). In the hydrolase group, 13 proteasome subunits were identified, accounting for 6.24% of EV proteins. Specifically, the most abundant proteins in EVs from *A. castellanii* were inosine-uridine-preferring nucleoside hydrolase family protein (IUNH) (13.92%), carboxylic ester hydrolase (4.76%), and peroxidase (4.25%). We found that EVs from *A. castellanii* contained approximately 5.5% proteins analogous to previously known exosomal proteins in other organisms, such as talin (0.23%), GM2 activator protein (0.23%), and filamin repeat domain-containing protein (0.16%). Together, these results suggest that numerous *A. castellanii*-specific proteins with enzymatic activity were packaged in EVs.

### Detection of the peptidase activities in EVs from *A. castellanii*

**Fig. 6:**
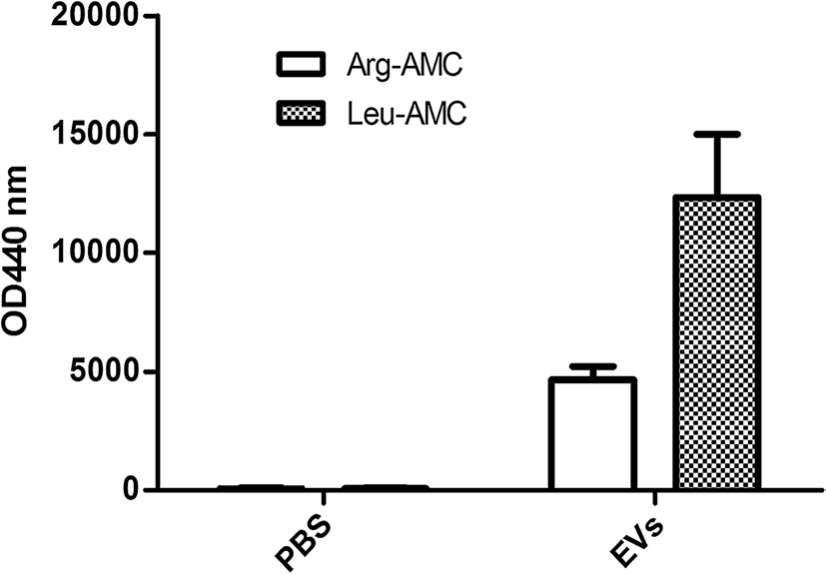
Detection of aminopeptidase activity in EVs from *Acanthamoeba castellanii*. The aminopeptidase activity of EVs was detected by the hydrolysis of Leu-AMC and Arg-AMC compared with that of the PBS-treated control. The released fluorescence with an emission wavelength of 440 nm represents the corresponding aminopeptidase activity

We previously reported that Asp can hydrolyze leucine and arginine, suggesting that aminopeptidases are included in the secreted fraction and that this may be important for disruption of host cells [15]. Based on the proteomic analysis of EVs from *A. castellanii*, there are several proteins with aminopeptidase activities (Additional file 2: Table S2), which may also contribute to the pathogenesis of *Acanthamoeba*. To validate the peptidase activities in EVs, we detected the levels of AMC-labeled arginine and leucine by a spectrophotometer (Fig. 6), which could reflect the activities of corresponding aminopeptidases. Compared with the PBS-treated control, the levels of AMC-labeled arginine and leucine were significantly increased following treatment with EVs from *A. castellanii*, confirming that the aminopeptidases were enriched in the proteome.

## Discussion

We successfully purified EVs from *A. castellanii* and demonstrated their potential roles in the pathogenesis of this parasite, highlighting the significance of EVs in destroying and delivering messages to the mammalian cells *via* contact-independent mechanisms in addition to secreted proteins [15]. Additionally, EVs from *A. castellanii* induced the immune response of THP-1 cells. The first quantitative proteomic analysis of EVs from *A. castellanii* was also conducted, providing an in-depth understanding of the abundance of molecules inside the EVs and their possible functions in mediating pathogenesis.

In a recent report, the EVs secreted by *A. castellanii* were isolated using a differential centrifugation approach [33]. These EVs grown in two nutritional conditions were characterized, including nutrient-rich PYG medium (PYG-EVs) and nutrient-deficient glucose medium (glucose-EVs). Although a different approach for EV isolation was used in the present study, we showed that the size of EVs (mean diameter = 166.7 nm) is similar to that in the previous study (117.1 ± 73.3 and 117.7 ± 55.8 nm for PYG-EVs and glucose-EVs, respectively). Specifically, we clearly identified the morphology of EVs with their double-layered membrane.

Monocytes/macrophages are considered to be important effectors in response to *Acanthamoeba* infections [41]; however, the biochemical mechanisms that occur in human monocytes upon exposure to *Acanthamoeba*-secreted products are far from understood. EVs often elicit a pro-inflammatory response, which enhances the parasite burden in the host [17]. To our knowledge, we showed for the first time that EVs from *A. castellanii* induce the host immune response, stimulating the expression of the pro-inflammatory cytokines IL-6 and IL-12 by human THP-1 cells. IL-6 is a chief stimulator of acute inflammation, and its production by the host appears to be a common immune response to EVs of various protozoans, such as *Trichomonas vaginalis*, *Plasmodium* spp. (infecting erythrocytes) and *Trypanosoma brucei* [17]. IL-12 induces the cytotoxic activity of phagocytes, the production of INF-γ by natural killer cells, and the development of cytotoxic lymphocytes [42]. A previous study demonstrated that the production of the cytokines IL-6 and IL-12 by THP-1 cells was significantly increased after coincubation with the soluble products contained in amoebic conditioned cell-free medium (aCM), whereas no marked elevation of these cytokines was observed after coincubation with *Acanthamoeba* trophozoites [34]. This *in vitro* observation leads us to hypothesize that the stimulation of such pro-inflammatory cytokine release by aCM is mediated, at least partially, through EVs. Future *in vivo* models developed to study the effects of *Acanthamoeba* EVs on various host effector cells recruited during infection will be necessary to understand the complicated immune response and its impact on disease progression.

It has been demonstrated that *A. castellanii* EVs display cytotoxicity to Chinese hamster ovary (CHO) and T98G mammalian cells *via* necrosis and apoptosis, respectively, suggesting that different cell death mechanisms are induced by EVs in various host cells [33, 35]. We also showed that EVs cause cell disruption and reduce cell adhesion ability, resulting in CPE on rat glial C6 cells, which is similar to the outcome exerted by the secreted proteins [15]. These *in vitro* findings suggest that EVs mediate the pathogenesis of *A. castellanii*. The secreted proteases have been shown to play a key role in the pathogenesis of several protozoans, such as *T. vaginalis* [43], *Giardia lamblia* [44], *Leishmania* spp. [45] and *Entamoeba histolytica* [46]; however, the proteases are mainly identified in the EV-free supernatant of *A. castellanii* [33]. Hence, other previously uncharacterized proteins with potential impact on virulence and biological functions in EVs from *A. castellanii* deserve further investigation. For example, several proteasome subunits have been identified in EVs of this species. It has been demonstrated that the proteasome is involved in the regulation of encystation and extracellular proteolytic activities of *Acanthamoeba* [47]. It is worthwhile to study whether the proteasome subunits in EVs are involved in *Acanthamoeba* differentiation or in cytotoxicity to host cells.

A previous study has identified 110 and 148 proteins in EVs from *A. castellanii* grown in PYG and glucose medium, respectively [33]. The majority of proteins in PYG-EVs and glucose-EVs belonged to the ribosomal and miscellaneous protein categories, respectively. Different protein compositions in PYG-EVs and glucose-EVs suggest that the proteins packaged in EVs were affected by different environmental cues. However, that report was a qualitative analysis of the protein composition in EVs of *A. castellanii*, which could not reflect the abundance of proteins. Herein, we conducted quantitative proteomic analysis of EVs and found that the largest protein families in EVs were categorized into the classes of hydrolases and oxidoreductases. In comparison with the previously identified proteins in PYG-EVs [33], 13 of the same proteins were identified in our study. The differences in EV proteome profiles identified from independent studies may be due to the use of different strains, isolation methods, and culture conditions. It is feasible to define a common set of *Acanthamoeba* exosome proteins derived from more studies and produce the specific antibodies as biomarkers to recognize the isolated exosomes. We note that only a small portion of proteins in EVs were analogous to previously identified EV proteins, suggesting that many distinct proteins are packed into the EVs of *A. castellanii*. For example, we showed that the most abundant protein in the EVs was the IUNH family protein, which catalyzes the hydrolysis of the N-glycosidic bond of all of the commonly occurring purine and pyrimidine nucleosides into ribose and the associated base [48]. IUNH is important for protozoan parasites incapable of *de novo* biosynthesis of purines, such as *Leishmania major* [49], exerting the function of purine salvage from the host. However, it has been shown that *A. polyphaga* is able to utilize radiolabeled formate and glycine as precursors for purine biosynthesis [50]. Hence, the biological significance of IUNH in free-living protozoans remains to be determined. Comparative proteomic analysis of proteins secreted into the extracellular space of pathogenic and non-pathogenic *A. castellanii* also revealed that IUNH is upregulated more than 5-fold in the pathogenic strain [51]. It is worth investigating whether IUNH in EVs plays a crucial role in the pathogenesis of *A. castellanii*.

## Conclusions

Collectively, our findings provide *in vitro* experimental evidence that EVs form *A. castellanii* are internalized into rat glial C6 cells, induce the immune response of the human monocyte/macrophage lineage, and cause the disruption of their target cells. Our quantitative proteomic analysis of EVs revealed several previously uncharacterized proteins, such as the proteasome subunits and IUNH, shedding new light on their potential roles during the establishment of *Acanthamoeba* infections.

## Supplementary information

**Additional file 1: Table S1.** Primers used in this study.

**Additional file 2: Table S2.** Identification of proteins in EVs from *A. castellanii* by quantitative proteomic analysis.

## Data Availability

Data supporting the conclusions of this article are included within the article and its additional files. The datasets used and/or analyzed during the present study will be made available by the corresponding author upon reasonable request. The mass spectrometry proteomics data have been deposited to the ProteomeXchange Consortium *via* PRIDE partner repository with the dataset identifier PXD014735.

## Abbreviations

EVs: extracellular vesicles
GAE: granulomatous amoebic meningoencephalitis
AK: *Acanthamoeba* keratitis
MIP133: mannose-induced 133 kDa protease
MVBs: multivesicular bodies
IL-6: interleukin-6
CPE: cytopathic effect
TNF-α: tumor necrosis factor alpha
Asp: *Acanthamoeba* secreted proteins
IUNH: inosine-uridine-preferring nucleoside hydrolase family protein
Leu-AMC: l-leucine-7-amido-4-methylcoumarin hydrochloride
Arg-AMC: l-arginine-AMC
PYG: peptone yeast extract glucose
ECM: extracellular matrix
aCM: amoebic conditioned cell-free medium
